# Patching of the Inferior Vena Cava Following Lateral Venorrhaphy in Penetrating Traumatic Injury

**DOI:** 10.1155/2022/5488752

**Published:** 2022-12-23

**Authors:** Jane Keating, Jason Wade, Katerina Dukleska, James Healy, Elizabeth Aitchinson, Jonathan Gates

**Affiliations:** ^1^Divisions of Acute Care Surgery and Vascular Surgery, Hartford Hospital, Hartford, CT, USA; ^2^University of Connecticut School of Medicine, Farmington, CT, USA; ^3^Thomas Jefferson University, Philadelphia, PA, USA; ^4^Division of Pediatric Surgery, Connecticut Children's Medical Center, Hartford, CT, USA

## Abstract

Penetrating injury to the inferior vena cava (IVC) is associated with high morbidity and mortality. Luminal narrowing can occur following lateral venorrhaphy and can lead to future morbidity. This case report discusses the success of patch repair following lateral venorrhaphy in two trauma patients. We describe the use of patch repair to eliminate stenosis of the IVC resulting from primary repair in the setting of traumatic injury. Furthermore, trauma patients are known to be at high risk for venous thromboembolism, and we describe the use of low molecular weight heparin as chemical prophylaxis for prevention of this complication following patch repair.

## 1. Introduction

Penetrating injury to the abdomen involving the major vasculature, including the inferior vena cava (IVC), is associated with high morbidity and mortality. Death prior to hospital arrival occurs in up to 50% of patients with IVC injury, and of those patients who arrive at a trauma center with signs of life, mortality occurs in over half of patients [1]. Injury to the IVC classically presents as hemorrhagic shock with a zone 1 hematoma upon abdominal exploration. These patients often undergo massive transfusion, have additional injuries to the abdominal viscera, and are frequently managed initially with a damage control laparotomy. The tenets of damage control include expeditious control of bleeding and contamination followed by temporary abdominal closure to allow for ongoing resuscitation prior to definitive re-exploration upon improved physiology [2–4]. Major vascular injuries often require exploration with prompt proximal and distal controls and direct repair of the vessel using running suture; however, this damage control repair often results in a luminal narrowing of the IVC.

Patients in extremis may undergo ligation of the IVC at the time of initial laparotomy [5, 6]. Research suggests that ligation of the IVC may be associated with negative outcomes including higher mortality and increased rates of acute kidney injury and pulmonary embolism (PE) [7, 8]. While some patients' physiology necessitates ligation, others may tolerate direct repair of the IVC in a running fashion during initial laparotomy. Those patients who undergo lateral venorrhaphy experience long-term complications related to luminal narrowing, including IVC thrombosis, lower extremity edema, deep vein thrombosis (DVT), and PE [9].

Our institution has had a positive experience with performing vein patching from cryopreserved cadaveric aortic tissue at the time of controlled operative take-back following damage control as described in the second case below. We suggest that this method when used to reduce IVC stenosis from primary damage control repair may reduce the incidence of IVC thrombus, deep venous thrombosis, lower extremity edema, and pulmonary embolus, and may result in overall improved outcomes. Below we discuss the technique used with two patients who suffered gunshot wounds to the abdomen resulting in multiple injuries including direct injury to the IVC. Both patients presented in March of 2021 to our urban level 1 trauma center and underwent initial damage control repair of the vein with obvious narrowing but were later definitively repaired using vein patching. In our opinion, IVC patching should be considered routinely in hemodynamically sufficient patients during early take-back definitive abdominal operations.

## 2. Case Description

### 2.1. Patient 1

A 33-year-old male was brought into our emergency department after suffering a single gunshot wound to his abdomen in the right upper quadrant. Upon arrival, his airway was intact, and his breath sounds were present; however, he was noted to be diaphoretic and hypotensive. A focused assessment with sonography in trauma (FAST) exam was positive, and a chest X-ray showed no hemopneumothorax or bullet within the thoracic cavity. A kidney, ureter, and bladder (KUB) plain film showed a retained bullet in the midline pelvis consistent with an intraabdominal trajectory. Large bore intravenous access was obtained, and two units of whole blood were administered on the way to the operating room.

Immediately upon entering his abdomen, we encountered moderate hemoperitoneum. He was noted to have a zone 1 inframesocolic hematoma on the right side. We performed Cattell-Braasch and Kocher maneuvers. As we dissected down into the hematoma, we first encountered a large hole in the lateral zone 2 duodenum through which the bullet had tracked. We quickly whip stitched this to control contamination. Next, we encountered copious dark red bleeding from the infrarenal IVC. We controlled this with a sponge stick applied directly to the anterior wound for temporary control. With proximal and distal controls, the injury was noted to be through and through with a 1 cm defect to both the anterior and posterior walls of the infrarenal IVC. The anterior injury was extended proximally and distally to allow for exposure of the back wall injury. The posterior injury was repaired first with running 4-0 Prolene suture from within the lumen of the IVC. When the posterior closure was completed, the anterior injury was repaired similarly. The lumen of the IVC was significantly narrowed with both the anterior and posterior suture lines, but a small lumen was present. Although he was well resuscitated, he was cold and coagulopathic. Given the extent of his injuries, we elected to continue in a damage control mode for the remainder of the surgery. His other injuries included an anti-mesenteric injury to the second portion of the duodenum, which had been temporarily closed with no apparent leak. He also had transverse colon and small bowel injuries, which were resected and left in discontinuity. A temporary abdominal vacuum closure was performed.

**Figure 1:**
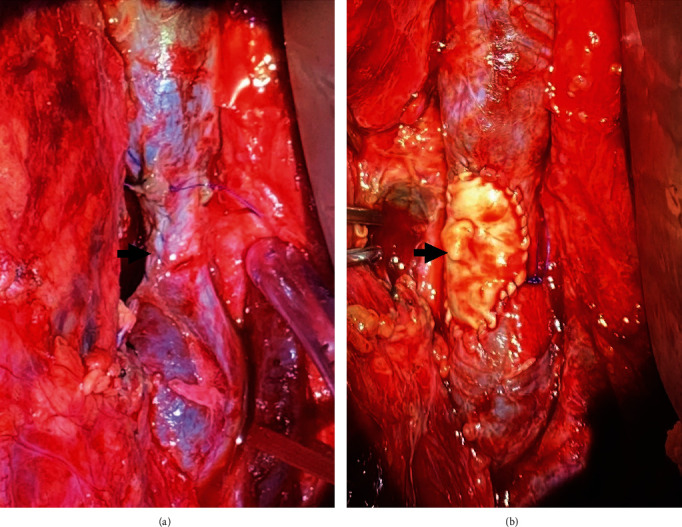
Intraoperative image of IVC: (a) Luminal narrowing status post lateral venorrhaphy prior to patch repair and (b) restored luminal diameter following patch repair.

On postoperative day 1, he returned to the operating room for planned re-exploration. Direct examination of the site of the IVC injury and repair indicated that there was clot within the IVC. Intraoperative ultrasound confirmed the clot in the IVC and documented that it extended down into the common iliac veins bilaterally. Control of the IVC both proximal and distal to the prior repair was completed with ligation of multiple posterior lumbar branches between silk ties or clips. Vessel loops were placed around the proximal IVC and common iliac veins bilaterally. Additionally, we obtained proximal and distal controls of the aorta and iliac arteries. With adequate proximal and distal venous controls, the patient was given an intravenous heparin bolus, and time allowed for adequate circulation. A clamp was placed on the proximal IVC just below the renal vein takeoff. The prior repair was opened, and fresh appearing thrombus was encountered. The origin of the clot was at the suture line repair with a tongue of fresh clot extending upward toward the renal veins that was clearly anchored distally but freely mobile more proximally. This was removed, and a #6 Fogarty was run down each iliac vein sequentially with removal of a significant amount of acute thrombus and restoration of brisk venous bleeding. A cryopreserved piece of femoral vein was thawed and prepared according to the protocol. A segment of this was opened longitudinally to serve as an on-lay patch. The prior posterior IVC wall repair was inspected and appeared intact. The cryopreserved femoral vein patch was then sewn to the anterior wall defect with 4-0 running Prolene suture. The repair was irrigated with heparinized saline and then vented prior to completion of the closure to ensure no air or additional thrombus was present. The clamps were then removed, and the repair appeared hemostatic. There was no longer narrowing of the vena cava (Figure 1). Intraoperative duplex confirmed a patent compressible lumen with no evidence of residual iliac vein thrombus.

The duodenum was repaired primarily in two layers, and a small bowel anastomosis was performed at the site of his previous injury. A tongue of omentum was positioned in between the IVC repair and the duodenal repair. A right colectomy was performed for the transverse colon injury, and an ileocolic anastomosis was performed. A Blake drain was positioned in the peri-duodenal area down into Morison's pouch, and a nasal Dobhoff tube was placed into the jejunum. His abdomen was closed, and he returned to the intensive care unit. Postoperatively he did well. He was placed on therapeutic enoxaparin. He was extubated postclosure day 1. His intraabdominal drain remained serosanguinous and negative for amylase. He underwent an upper gastrointestinal barium study, which was negative for duodenal leak five days following abdominal closure. He experienced a transient ileus, which was managed conservatively. He tolerated a diet and was discharged on hospital day 10. He has since suffered no lower extremity edema and has screened negative for DVT with bilateral lower extremity duplexes prior to clinic follow-up 2 weeks following discharge. The enoxaparin was stopped after three months of therapy.

### 2.2. Patient 2

A 15-year-old male was brought into our emergency department following a single gunshot wound to the left upper quadrant abdomen. Upon arrival, he was protecting his airway, and his breath sounds were intact bilaterally; however, he was hypotensive. A FAST exam was positive for intraabdominal blood. His chest X-ray showed no hemopneumothorax or retained bullet. A bullet was retained in his right abdomen on KUB. Massive transfusion was initiated, and he was brought to the operating room for emergency laparotomy.

Upon entering his abdomen, he was noted to have a moderate amount of blood in his abdomen. A zone 1 retroperitoneal hematoma was identified as well as a hematoma at the base of the small bowel mesentery. Exploration of the root of the mesentery revealed a direct injury to a substantial proximal jejunal venous branch draining into the superior mesenteric vein. This branch was dissected out and ligated. A right-sided medial visceral rotation was performed, which exposed brisk venous bleeding from an infrarenal IVC injury. This was controlled with a vascular clamp proximally and sponge-stick compression distally, whereas a running 4-0 Prolene suture was used to close the defect in the vein. This repair resulted in hemostasis, but with substantial narrowing of the venous lumen and pre-stenotic dilation. In addition to this injury, he suffered injury to the third portion of the duodenum, the mid-jejunum, the transverse colon, and the right ureter. Due to hemodynamic instability and damage control nature of the initial procedure, the decision was made to defer patch angioplasty. The bowel injuries were resected, and he was left in discontinuity with a temporary abdominal closure.

**Figure 2:**
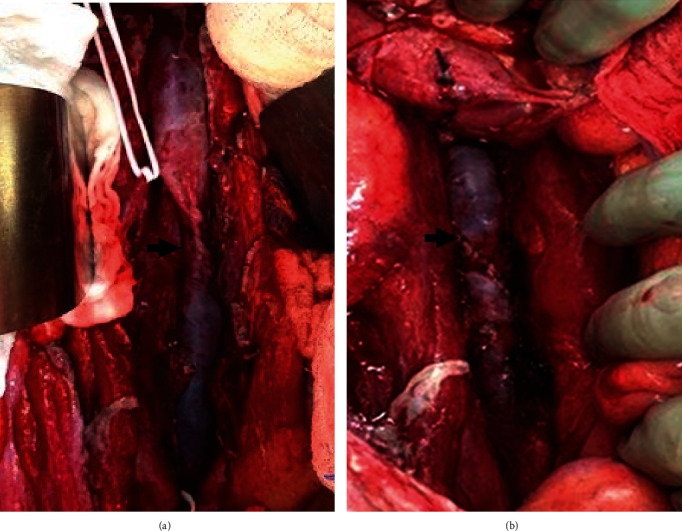
Intraoperative image of IVC: (a) luminal narrowing status post lateral venorrhaphy prior to patch repair and (b) restored luminal diameter following patch repair.

The following day, the patient's hemodynamic instability, hypothermia, and coagulopathy had been corrected, and he returned to the operating room in standard fashion for re-exploration. Due to concern for IVC narrowing during the index operation, the preoperative plan included cadaveric homograft patch angioplasty of the IVC. The abdomen was re-entered, and as suspected, the IVC repair appeared stenotic as previously described. Proximal and distal controls were obtained with vessel loops and then replaced with low profile vascular clamps. The IVC was then re-opened by carefully removing the prior suture repair, and the patient was bolused with heparin. There was no clot in the IVC. Next, the cadaveric aortic homograft patch was cut into an ellipse that would cover the size of the defect and sutured in a running watertight fashion using 4-0 Prolene. This resolved the pre-stenotic dilation (Figure 2). He was maintained on a heparin infusion postoperatively and transitioned to therapeutic enoxaparin when stable from his other injuries.

Doppler ultrasound of the lower extremities 1 week following his repair demonstrated no evidence of DVT. He ultimately underwent pyloric exclusion with duodeno-jejunostomy and gastrojejunostomy, right hemicolectomy with end ileostomy, and percutaneous nephrostomy and trans-ureteral stenting to manage his additional injuries. He did well postoperatively and was discharged home on postoperative day 15. Enoxaparin was continued for three months.

## 3. Discussion

We have performed several IVC patches following penetrating injury to the abdomen over the last three years with favorable outcomes. In this study, we provide discussion of two such patients. Both patients had several additional injuries requiring resuscitation with balanced blood products and damage control laparotomy. Both patients did well with no complications related to the venous patch procedure. We recommend and consider IVC patching for similar patients with stenosis following repair and typically during the take-back following damage control surgery. While patching options include prosthetic and autogenous patching, we did use cadaveric aortic tissue for our patching in the second case described here [10]. We prefer this approach as it limits the negative effects of prosthetic material in the case of infection, allows tailoring of the patch to the size of the defect, and does not expose the patient to additional risks associated with vein harvesting. That said, autologous patching with vein is perfectly acceptable. We have a low threshold for using autologous vein to repair many vascular injuries, but on occasion in which the IVC injury is extensive, the size of the patch needed might exceed that of a saphenous vein that had been fashioned into a patch without spending the time to create a panel graft. This is especially true in the case of anterior and posterior injuries of the IVC.

There are no formal guidelines regarding the indications for postoperative systemic anticoagulation after IVC reconstruction for either oncologic or traumatic indications, and the perioperative risk of acute venous thromboembolic events (VTE) after IVC repair has not been well described. Hicks et al. describe their experience with 65 patients undergoing IVC reconstruction (primary repair, 25%; patch, 43%; and graft, 32%) following surgery for cancer [11]. The overall incidence of VTE in these patients was 22% (DVT in 9% and PE in 12%). There was no significant difference between repair type and VTE event. Although this experience is not among trauma patients, trauma patients, like cancer patients, suffer a high rate of VTE. Similar to oncology patients, trauma patients have been shown to experience increased risk of VTE [12]. While later studies have suggested a lesser incidence of thrombotic events [13, 14], the benefit of VTE prophylaxis is well established [12, 15]. Despite the limited guidance, we recommend three months of anticoagulation postrepair using therapeutic enoxaparin for trauma patients. Although data to support this are lacking, we have had favorable results using this approach. If the patient is unable to tolerate anticoagulation secondary to additional injuries, the patient can be treated with aspirin 325 mg two times a day or without anticoagulation.

Future research is needed to compare outcomes for patients treated with patching, primary repair, and ligation on a larger scale. Additionally, among patients that undergo IVC patching, future research should aim to determine optimal strategy regarding both patch type and postoperative anticoagulation strategies.

## 4. Conclusion

Here, we describe two cases of penetrating trauma resulting in injury to the IVC. Lateral venorrhaphy performed at initial damage control laparotomy may result in luminal narrowing of the IVC as demonstrated in the cases described. Patch repair at abdominal re-exploration allowed for restoration of venous diameter. Postoperative low molecular weight heparin chemical prophylaxis was used to prevent VTE and is our recommendation in this high risk population.

## Data Availability

All data regarding this case report has been reported in the manuscript. Please contact the corresponding author in case of requiring any further information.
